# Assessment of Racial/Ethnic Disparities in Hospitalization and Mortality in Patients With COVID-19 in New York City

**DOI:** 10.1001/jamanetworkopen.2020.26881

**Published:** 2020-12-04

**Authors:** Gbenga Ogedegbe, Joseph Ravenell, Samrachana Adhikari, Mark Butler, Tiffany Cook, Fritz Francois, Eduardo Iturrate, Girardin Jean-Louis, Simon A. Jones, Deborah Onakomaiya, Christopher M. Petrilli, Claudia Pulgarin, Seann Regan, Harmony Reynolds, Azizi Seixas, Frank Michael Volpicelli, Leora Idit Horwitz

**Affiliations:** 1 Department of Population Health, New York University Grossman School of Medicine, New York; 2 Department of Medicine, New York University Grossman School of Medicine, New York; 3 Associate Editor, *JAMA Network Open*; 4 Department of Psychiatry, New York University Grossman School of Medicine, New York

## Abstract

**Importance:**

Black and Hispanic populations have higher rates of coronavirus disease 2019 (COVID-19) hospitalization and mortality than White populations but lower in-hospital case-fatality rates. The extent to which neighborhood characteristics and comorbidity explain these disparities is unclear. Outcomes in Asian American populations have not been explored.

**Objective:**

To compare COVID-19 outcomes based on race and ethnicity and assess the association of any disparities with comorbidity and neighborhood characteristics.

**Design, Setting, and Participants:**

This retrospective cohort study was conducted within the New York University Langone Health system, which includes over 260 outpatient practices and 4 acute care hospitals. All patients within the system’s integrated health record who were tested for severe acute respiratory syndrome coronavirus 2 between March 1, 2020, and April 8, 2020, were identified and followed up through May 13, 2020. Data were analyzed in June 2020. Among 11 547 patients tested, outcomes were compared by race and ethnicity and examined against differences by age, sex, body mass index, comorbidity, insurance type, and neighborhood socioeconomic status.

**Exposures:**

Race and ethnicity categorized using self-reported electronic health record data (ie, non-Hispanic White, non-Hispanic Black, Hispanic, Asian, and multiracial/other patients).

**Main Outcomes and Measures:**

The likelihood of receiving a positive test, hospitalization, and critical illness (defined as a composite of care in the intensive care unit, use of mechanical ventilation, discharge to hospice, or death).

**Results:**

Among 9722 patients (mean [SD] age, 50.7 [17.5] years; 58.8% women), 4843 (49.8%) were positive for COVID-19; 2623 (54.2%) of those were admitted for hospitalization (1047 [39.9%] White, 375 [14.3%] Black, 715 [27.3%] Hispanic, 180 [6.9%] Asian, 207 [7.9%] multiracial/other). In fully adjusted models, Black patients (odds ratio [OR], 1.3; 95% CI, 1.2-1.6) and Hispanic patients (OR, 1.5; 95% CI, 1.3-1.7) were more likely than White patients to test positive. Among those who tested positive, odds of hospitalization were similar among White, Hispanic, and Black patients, but higher among Asian (OR, 1.6, 95% CI, 1.1-2.3) and multiracial patients (OR, 1.4; 95% CI, 1.0-1.9) compared with White patients. Among those hospitalized, Black patients were less likely than White patients to have severe illness (OR, 0.6; 95% CI, 0.4-0.8) and to die or be discharged to hospice (hazard ratio, 0.7; 95% CI, 0.6-0.9).

**Conclusions and Relevance:**

In this cohort study of patients in a large health system in New York City, Black and Hispanic patients were more likely, and Asian patients less likely, than White patients to test positive; once hospitalized, Black patients were less likely than White patients to have critical illness or die after adjustment for comorbidity and neighborhood characteristics. This supports the assertion that existing structural determinants pervasive in Black and Hispanic communities may explain the disproportionately higher out-of-hospital deaths due to COVID-19 infections in these populations.

## Key Points

**Question:**

Do outcomes among patients with coronavirus disease 2019 (COVID-19) differ by race/ethnicity, and are observed disparities associated with comorbidity and neighborhood characteristics?

**Findings:**

This cohort study including 9722 patients found that Black and Hispanic patients were more likely than White patients to test positive for COVID-19. Among patients hospitalized with COVID-19 infection, Black patients were less likely than White patients to have severe illness and to die or be discharged to hospice.

**Meaning:**

Although Black patients were more likely than White patients to test positive for COVID-19, after hospitalization they had lower mortality, suggesting that neighborhood characteristics may explain the disproportionately higher out-of-hospital COVID-19 mortality among Black individuals.

## Teaser

This cohort study of patients testing positive for COVID-19 in a large New York City health system compares rates of hospitalization, critical illness, mortality across racial/ethnic categories.

## Introduction

Reports across the US suggest that Black and Hispanic populations have higher coronavirus disease 2019 (COVID-19)–related hospitalization and mortality than White populations. According to the US Centers for Disease Control and Prevention, Black and Hispanic populations in major cities like New York City (NYC) account for a disproportionate share of COVID-19–related mortality relative to their representation in the population.^1,2^

The racial/ethnic disparities in COVID-related mortality may be explained by increased risk of disease because of difficulty engaging in social distancing because of crowding and occupation, and increased disease severity because of reduced access to health care, delay in seeking care,^3^ or receipt of care in low-resourced settings.^4^ Another explanation may be the higher rates of hypertension, diabetes, obesity, and chronic kidney disease (CKD) among Black and Hispanic populations, all of which worsen outcomes.^5,6,7,8,9,10,11^ The role of comorbidity in explaining racial/ethnic disparities in hospitalization and mortality has been investigated in only 1 study,^12^ which did not include Hispanic patients. Although poverty, low educational attainment, and residence in areas with high densities of Black and Hispanic populations are associated with higher hospitalizations and COVID-19–related deaths in NYC,^13^ the effect of neighborhood socioeconomic status (SES) on likelihood of hospitalization, severity of illness, and death is unknown. COVID-19–related outcomes in Asian patients have also been incompletely explored.

New York City, with over 231 824 reported cases and 19 153 deaths as of September 22, 2020,^14^ is one of the hardest hit areas in the US. In a large cohort of patients tested for COVID-19 at New York University Langone Health (NYULH) system, we compared outcomes (ie, positive test, hospitalization, critical illness, death) based on race and ethnicity, and assessed the association of any disparities with comorbidity and neighborhood SES.

## Methods

### Study Setting and Design

This study was conducted at the NYULH system, which includes over 260 outpatient office sites and 4 acute care hospitals ranging from a quaternary care hospital to a safety-net hospital. We identified all patients in NYULH’s integrated electronic health record (EHR) tested for COVID-19 between March 1, 2020, and April 8, 2020, and studied them through May 13, 2020. Data were analyzed in June 2020. Patients missing all data except age and sex who had no prior visits and no hospitalization were excluded. Patients who could not be geocoded to a Census tract were also excluded. This study follows the Strengthening the Reporting of Observational Studies in Epidemiology (STROBE) reporting guideline for reporting observational studies. This study was approved by the NYU Grossman School of Medicine institutional review board, which granted both a waiver of informed consent (as a minimal-risk medical record review) and a waiver of the Health Information Portability and Privacy Act.

### Outcomes: Testing Positive, Hospitalization, and Critical Illness

Testing was performed for patients presenting to outpatient offices, employee health offices, or the emergency department as clinically indicated. COVID-19 was defined as a positive result on real-time reverse-transcriptase–polymerase chain reaction assay. Patients were classified as hospitalized if they were admitted to NYULH after testing positive for COVID-19. Critical illness was defined as a composite of care in the intensive care unit, use of mechanical ventilation, discharge to hospice, or death. For patients with multiple encounters, the most severe outcome that occurred across encounters was assigned.

### Predictor Variables: Race and Ethnicity

Race and ethnicity were categorized using self-reported EHR data. Patients who self-reported Hispanic ethnicity were classified as Hispanic regardless of race. If ethnicity was missing, self-reported race was assigned. For patients with multiple entries for race, the entry with the most information was chosen. For example, if a patient had their race recorded as unknown in one clinical encounter and Black in another, that individual was classified as Black. Patients who reported more than 1 race (eg, White and Asian), were categorized as multiracial/other. Patients missing both ethnicity and race were classified as unknown. This coding process resulted in 6 groups: non-Hispanic White, non-Hispanic Black, Hispanic, Asian, multiracial/other, and unknown.

### Covariates: Patient Characteristics, Comorbidity, and Socioeconomic Status

For patients with COVID-19 test results, we extracted EHR data including age, sex, obesity (defined by body mass index [BMI] >30 [calculated as weight in kilograms divided by height in meters squared]), history of smoking (current, former, or never), and medical comorbidity (diabetes, hypertension, hyperlipidemia, coronary artery disease, CKD, heart failure, chronic obstructive pulmonary disease or asthma, and cancer). Comorbidity was defined based on diagnostic codes in the EHR. Vital signs and laboratory results obtained upon admission were also extracted where available.

Neighborhood SES was determined by geocoding home addresses and zip codes in the EHR and matching to census tracts using ArcGIS software version 10.0 (ESRI). Confidence of the geocoding accuracy was classified, and only matches with high confidence of accuracy were maintained for this analysis; addresses were then spatially joined to census tracts.^15^ The Agency for Healthcare Research and Quality (AHRQ) SES Index was calculated using American Community Survey data to assign an SES score to each patient.^16^ The AHRQ SES index is a validated, weighted composite of 7 indicators, including: percentage of population within a patient’s census tract in the labor force who are unemployed; percentage living below poverty level; median household income; median value of owner-occupied dwellings; percentage of residents aged 25 years or older with less than a 12th grade education; percentage who are aged 25 years or older completing 4 or more years of college; and the percentage of households that average 1 or more persons per room.^17,18^ This methodology was previously used in NYC to augment EHR data.^17^

### Statistical Analysis

Descriptive statistics were used to characterize each racial/ethnic group of patients: COVID-test result status (ie, negative and positive), hospitalization status, and with or without critical illness (ie, received care in the ICU, on mechanical ventilation, discharged to hospice, or died). Demographic and clinical characteristics were described and then stratified by race and ethnicity. Multivariable logistic regression models were conducted to identify factors independently associated with the following outcomes: testing positive for COVID-19, hospitalization, and critical illness. Competing risk survival analyses for the outcome of mortality or discharge to hospice, where discharge home alive was the competing risk with time from first positive test as the start point, were constructed including only hospitalized patients. Patients still hospitalized as of May 13, 2020, were counted as censored. The model was fitted with the R library cmprisk^19^ and the proportionality assumption was checked with the goffte library.^20^ All selected exposures (race/ethnicity) and covariates (patient characteristics, comorbidities, neighborhood SES) were included based on a priori clinical significance after testing for collinearity using the variance inflation factor to ensure none had a quotient above 2.^21^

For the model with testing positive for COVID-19 as outcome, all patients who had COVID-19 test results were included. For the model with hospitalization as the outcome, only patients who tested positive for COVID-19 were included. The critical illness model excluded 4 patients who were still hospitalized as of May 13, 2020, and had not met criteria for critical illness because the final outcome of hospitalization was not yet determined. Odds ratios (ORs) were obtained from each model, and confidence intervals for the ORs were bootstrapped using the approach of Venables and Ripley^22^ because assuming normality of the maximum likelihood estimate to estimate confidence intervals can be biased.^23^ All logistic regression models were conducted with R, version 3.6.3 (R Project for Statistical Computing). All analyses used 2-sided statistical tests and a *P* value <.05 was considered to be statistically significant.

## Results

### Characteristics of the Study Population

During the study period, 11 547 patients were tested for COVID-19, of whom 918 (8.0% [631 negative, 287 positive]) were excluded for lack of clinical data and 907 (7.9% [461 negative, 446 positive]) were excluded whose addresses could not be geocoded. Of the remaining 9722 patients (mean [SD] age 50.7 [17.5] years; 58.8% women), 4843 (49.8%) tested positive; of the COVID-19–positive patients, 2623 (54.2%) were admitted to an NYULH hospital (see eTable in Supplement for more details on patient demographic information). Among the 2623 patients hospitalized, 1858 (70.8%) were discharged alive; 36.3% experienced critical illness; 24.7% died or were discharged to hospice; and 4.5% remained hospitalized as of May 13, 2020 (see eFigure in the Supplement). Table 1 shows the proportion of COVID-19–positive patients by race/ethnicity and the outcomes by race/ethnicity among COVID-19–positive patients who were hospitalized.

**Table 1.** Proportion and Outcomes of Patients With COVID-19 by Race/Ethnicity

Characteristics	Patients, No. (%)						
	Total	White, non-Hispanic	Black, non-Hispanic	Hispanic	Asian, non-Hispanic	Multiracial/other, non-Hispanic	Unknown
COVID-19 positive status by race							
Total, No.	9722	4187	1353	2087	831	714	550
Positive	4843 (49.8)	1887 (45.1)	759 (56.1)	1255 (60.1)	341 (41.0)	358 (50.1)	243 (44.2)
Not positive	4879 (50.2)	2300 (54.9)	594 (43.9)	832 (39.9)	490 (59.0)	356 (49.9)	307 (55.8)
Outcomes among COVID-19–positive hospitalized patients by race							
Total, No.	2623	1047	375	715	180	207	99
Critical illness^a^	951 (36.3)	420 (40.1)	107 (28.5)	232 (32.4)	77 (42.8)	77 (37.2)	38 (38.4)
Mechanical ventilation	624 (23.7)	239 (22.8)	66 (17.6)	178 (24.9)	62 (34.4)	53 (25.6)	26 (26.3)
ICU	625 (23.8)	251 (24)	75 (20.0)	164 (22.9)	51 (28.3)	56 (27.1)	28 (28.3)
Death/hospice	647 (24.7)	300 (28.7)	71 (18.9)	154 (21.5)	51 (28.3)	47 (22.7)	24 (24.2)
Discharged alive	1858 (70.8)	702 (67)	293 (78.1)	520 (72.7)	119 (66.1)	152 (73.4)	72 (72.7)
Still hospitalized	118 (4.5)	45 (4.2)	11 (2.9)	41 (5.7)	10 (5.6)	8 (3.8)	3 (3.0)

Abbreviation: ICU, intensive care unit.

^a^ Critical illness includes ICU stay, mechanical ventilation, and discharge to hospice or in-hospital death.

### Characteristics of Patients Testing Positive for COVID-19 by Race and Ethnicity

Patient characteristics (age, sex, BMI, smoking history, insurance status), comorbidity, and the AHRQ SES index of the 4843 patients (1887 [39.0%] White, 759 [15.7%] Black, 1255 [25.9%] Hispanic, 341 [7.0%] Asian, and 358 [7.4%] multiracial/other) who tested positive are shown in (Table 2). Overall, White patients were oldest, and Hispanic patients youngest (eg, age ≥75 years: 400 [21.2%] White patients vs 106 [8.4%] Hispanic patients). Black patients had the highest proportion of women patients (471 [62.1%]) compared with the other groups (range, 46.8%-51.0%). A total of 288 (4.7%) of the patients identified as self-pay or uninsured, with the lowest rates for White patients (54 [2.9%]) and similar rates for all other groups (range, 5.0%-6.2%). Black patients had comparable rates of Medicaid insurance as White patients (72 [9.5%] vs 163 [8.6%]), while Hispanic patients had the highest rate of Medicaid coverage (400 [31.9%]). White patients had the highest median neighborhood SES index (56.6; 95% CI, 52.6-60.6), followed by Asian (54.4; 95% CI, 49.8-58.9), Black (52.0; 95% CI, 48.3-54.7), and Hispanic (49.9; 95% CI, 46.0-54.0) patients. There were several between-group differences in comorbid conditions that have been associated with COVID-19 illness (Table 2). Among patients who tested positive, Black patients had the highest prevalence of hypertension and CKD (eg, CKD: 101 [13.3%] Black patients vs 146 [7.7%] White patients), while Hispanic and Black patients had the highest prevalence of diabetes (363 [28.9%] Hispanic patients vs 364 [19.3%] White patients). Compared with White patients, Asian patients had lower prevalence of each comorbidity except diabetes.

**Table 2.** Patient Characteristics, Socioeconomic Status, and Comorbidity by Race/Ethnicity Among 4843 COVID-19–Positive Patients

Characteristics	Patients, No. (%)					
	White, non-Hispanic (n = 1887)	Black, non-Hispanic (n = 759)	Hispanic (n = 1255)	Asian, non-Hispanic (n = 341)	Multiracial/other, non-Hispanic (n = 358)	Unknown (n = 243)
Age, y						
19-44	461 (24.4)	253 (33.3)	512 (40.8)	128 (37.5)	116 (32.4)	124 (51)
45-54	265 (14)	144 (19)	262 (20.9)	56 (16.4)	72 (20.1)	30 (12.3)
55-64	377 (20)	169 (22.3)	232 (18.5)	73 (21.4)	82 (22.9)	43 (17.7)
65-74	384 (20.3)	111 (14.6)	143 (11.4)	44 (12.9)	51 (14.2)	25 (10.3)
≥75	400 (21.2)	82 (10.8)	106 (8.4)	40 (11.7)	37 (10.3)	21 (8.6)
Sex						
Women	884 (46.8)	471 (62.1)	615 (49.0)	174 (51.0)	165 (46.1)	119 (49.0)
Men	1003 (53.2)	288 (37.9)	640 (51.0)	167 (49)	193 (53.9)	124 (51.0)
Smoking status						
Current	115 (6.1)	44 (5.8)	71 (5.7)	11 (3.2)	22 (6.1)	9 (3.7)
Former	460 (24.4)	110 (14.5)	184 (14.7)	30 (8.8)	55 (15.4)	30 (12.3)
Never	1135 (60.1)	518 (68.2)	811 (64.6)	231 (67.7)	221 (61.7)	163 (67.1)
Unknown	177 (9.4)	87 (11.5)	189 (15.1)	69 (20.2)	60 (16.8)	41 (16.9)
Insurance type						
Commercial	921 (48.8)	454 (59.8)	500 (39.8)	201 (58.9)	188 (52.5)	137 (56.4)
Medicaid	163 (8.6)	72 (9.5)	400 (31.9)	45 (13.2)	64 (17.9)	39 (16)
Medicare	676 (35.8)	178 (23.5)	232 (18.5)	67 (19.6)	76 (21.2)	40 (16.5)
Other	73 (3.9)	16 (2.1)	45 (3.6)	11 (3.2)	10 (2.8)	7 (2.9)
Uninsured/self-pay	54 (2.9)	39 (5.1)	78 (6.2)	17 (5.0)	20 (5.6)	20 (8.2)
BMI group						
<25	559 (29.6)	128 (16.9)	251 (20.0)	170 (49.9)	97 (27.1)	70 (28.8)
25 to <30	654 (34.7)	240 (31.6)	424 (33.8)	109 (32)	130 (36.3)	76 (31.3)
30 to <40	516 (27.3)	305 (40.2)	438 (34.9)	46 (13.5)	96 (26.8)	60 (24.7)
≥40	106 (5.6)	64 (8.4)	100 (8.0)	2 (0.6)	19 (5.3)	9 (3.7)
Unknown BMI	52 (2.8)	22 (2.9)	42 (3.3)	14 (4.1)	16 (4.5)	28 (11.5)
BMI, median (IQR)	28.5 (25-32.9)	30.6 (26.5-35.6)	29.8 (26.2-34.3)	24.8 (22.7-27.6)	28.1 (24.8-32.1)	27.3 (24.5-31.2)
Neighborhood SES, median (IQR)	56.6 (52.6-60.6)	52.0 (48.3-54.7)	49.9 (46.0-54.0)	54.4 (49.8-58.9)	53.6 (49.6-57.5)	54.9 (49.9-59.6)
Neighborhood SES, median (IQR), %						
Below federally defined poverty line	10.1 (5.2-19.5)	15.3 (8.2-24.5)	21.1 (11.0-29.7)	13.1 (6.5-22.9)	14.3 (7.6-24)	12.5 (6.6-22.4)
Aged ≥16 y or in labor force, unemployed, and actively seeking work	5.8 (4.0-8.2)	5.6 (2.1-10.5)	7.2 (4.6-10.4)	6.3 (4.0-9.1)	6.3 (4.0-9.9)	5.4 (3.6-8.0)
Households with ≥1 person per room	4.4 (1.5-10.3)	7.8 (4.1-11.5)	10.8 (5.1-18.3)	6.4 (2.2-14.6)	7.2 (3-12)	5.8 (2.5-11.8)
Aged ≥25 y with less than a 12th grade education	9.4 (4.7-17.5)	18.5 (12.1-25.2)	25.4 (14.7-36.3)	14.1 (6.9-29.9)	15.1 (7.8-26.2)	13.4 (6.3-23.4)
Aged ≥25 y with at least 4 y of college	40.7 (30.5-62.6)	24.4 (16.8-33.2)	22.7 (15.3-34.7)	35.9 (23.8-53.3)	32.8 (21.1-48.3)	37 (22.5-56.2)
Median household income (standardized to 0-100)^a^	28.6 (18.2-41.0)	22.2 (15.5-30.5)	19.7 (13.5-27.1)	25.3 (17.4-37.3)	22.9 (16.3-33.8)	25.6 (17.1-39.3)
Median value of owner-occupied homes (standardized to 0-100)^a^	28.3 (21.3-36.8)	18.5 (14.6-23.1)	22.4 (15.5-32.0)	26.2 (19.0-34.0)	25.7 (19.3-35.1)	24.8 (18.1-34.0)
Cardiovascular conditions						
Hypertension	830 (44.0)	399 (52.6)	465 (37.1)	131 (38.4)	121 (33.8)	50 (20.6)
Hyperlipidemia	798 (42.3)	232 (30.6)	395 (31.5)	100 (29.3)	83 (23.2)	42 (17.3)
Coronary artery disease	259 (13.7)	40 (5.3)	75 (6)	20 (5.9)	25 (7)	7 (2.9)
Heart failure	139 (7.4)	44 (5.8)	48 (3.8)	6 (1.8)	9 (2.5)	6 (2.5)
Diabetes	364 (19.3)	218 (28.7)	363 (28.9)	90 (26.4)	67 (18.7)	41 (16.9)
Cancer	202 (10.7)	55 (7.2)	68 (5.4)	27 (7.9)	26 (7.3)	11 (4.5)
CKD	146 (7.7)	101 (13.3)	114 (9.1)	23 (6.7)	21 (5.9)	6 (2.5)

Abbreviations: BMI, body mass index (calculated as weight in kilograms divided by height in meters squared); CKD, chronic kidney disease; IQR, interquartile range; SES, socioeconomic status.

^a^ Scale derived from the American Community Survey.

### Risk of Testing Positive for COVID-19 Among Patients Tested

In unadjusted models, Black (OR, 1.6; 95% CI, 1.4-1.8) and Hispanic (OR, 1.8; 95% CI, 1.6-2.0) patients were more likely than White patients to test positive for COVID-19. After adjusting for age, sex, insurance, and comorbidity, the risk was attenuated slightly for Hispanic patients (OR, 1.7; 95% CI, 1.5-1.9), but not for Black patients (OR, 1.6; 95% CI, 1.4-1.9). Addition of neighborhood SES to the model modestly reduced the risk for both Black (OR, 1.3; 95% CI, 1.2-1.6) and Hispanic patients (OR, 1.5; 95% CI, 1.3-1.7). The lower risk in Asian patients in unadjusted model (OR, 0.9; 95% CI, 0.8-1.0) disappeared after full adjustment (Table 3).

**Table 3.** Patient Characteristics, Socioeconomic Status, and Comorbidity by Race/Ethnicity Among All Hospitalized Patients

Characteristics	Patients, No. (%)					
	White, non-Hispanic (n = 1047)	Black, non-Hispanic (n = 375)	Hispanic (n = 715)	Asian, non-Hispanic (n = 180)	Multiracial/other, non-Hispanic (n = 207)	Unknown (n = 99)
Age, y						
19-44	104 (9.9)	53 (14.1)	182 (25.5)	27 (15.0)	32 (15.5)	17 (17.2)
45-54	102 (9.7)	56 (14.9)	155 (21.7)	27 (15.0)	38 (18.4)	12 (12.1)
55-64	188 (18.0)	102 (27.2)	155 (21.7)	48 (26.7)	60 (29.0)	28 (28.3)
65-74	281 (26.8)	88 (23.5)	120 (16.8)	39 (21.7)	43 (20.8)	21 (21.2)
≥75	372 (35.5)	76 (20.3)	103 (14.4)	39 (21.7)	34 (16.4)	21 (21.2)
Sex						
Women	393 (37.5)	188 (50.1)	266 (37.2)	73 (40.6)	73 (35.3)	26 (26.3)
Men	654 (62.5)	187 (49.9)	449 (62.8)	107 (59.4)	134 (64.7)	73 (73.7)
Smoking status						
Current	61 (5.8)	22 (5.9)	29 (4.1)	6 (3.3)	11 (5.3)	4 (4)
Former	288 (27.5)	76 (20.3)	123 (17.2)	16 (8.9)	36 (17.4)	9 (9.1)
Never	578 (55.2)	224 (59.7)	447 (62.5)	110 (61.1)	113 (54.6)	57 (57.6)
Unknown	120 (11.5)	53 (14.1)	116 (16.2)	48 (26.7)	47 (22.7)	29 (29.3)
Insurance type						
Commercial	271 (25.9)	141 (37.6)	123 (17.2)	60 (33.3)	61 (29.5)	20 (20.2)
Medicaid	119 (11.4)	50 (13.3)	305 (42.7)	39 (21.7)	57 (27.5)	33 (33.3)
Medicare	600 (57.3)	164 (43.7)	209 (29.2)	64 (35.6)	68 (32.9)	35 (35.4)
Other	47 (4.5)	15 (4.0)	42 (5.9)	10 (5.6)	9 (4.3)	4 (4.0)
Uninsured/self pay	10 (1.0)	5 (1.3)	36 (5.0)	7 (3.9)	12 (5.8)	7 (7.1)
BMI group						
<25	250 (23.9)	73 (19.5)	141 (19.7)	89 (49.4)	50 (24.2)	20 (20.2)
25 to <30	376 (35.9)	113 (30.1)	236 (33)	59 (32.8)	75 (36.2)	37 (37.4)
30 to <40	338 (32.3)	149 (39.7)	262 (36.6)	22 (12.2)	60 (29.0)	30 (30.3)
≥40	70 (6.7)	37 (9.9)	59 (8.3)	1 (0.6)	13 (6.3)	4 (4.0)
Unknown BMI	13 (1.2)	3 (0.8)	17 (2.4)	9 (5.0)	9 (4.3)	8 (8.1)
BMI, median (IQR)	28.9 (25.5-33.3)	30.7 (26.5-36.4)	29.9 (26.2-34.6)	24.8 (22.7-27.7)	28.3 (25-32.6)	27.8 (25.2-31.7)
Neighborhood SES, median (IQR)	54.9 (50.5-58.9)	52.5 (48.5-55.1)	49.5 (46-53.8)	52.9 (48.2-58.0)	53.1 (49.3-56.3)	52.9 (47.0-58.0)
Neighborhood SES, median (IQR), %						
Below federally defined poverty line	13.1 (5.8-22.5)	15.1 (7.7-24.0)	22.2 (12.6-31.2)	14.6 (8.2-26.3)	15.6 (9.1-24.4)	15.2 (7.1-27.1)
Aged ≥16 y or in labor force, unemployed & actively seeking work	6.0 (4.1-8.4)	5.3 (2.7-9.4)	7.3 (4.6-10.4)	6.3 (4.1-8.8)	6.3 (4.0-9.9)	5.8 (4.1-9.1)
Households with ≥1 person per room	6.1 (2.2-13.1)	7.1 (3.6-10.3)	11.9 (5.6-20.3)	9.6 (2.9-17.0)	8.7 (3.7-13.4)	8.6 (2.8-18.3)
Aged ≥25 y with less than a 12th grade education	12.1 (6.4-20.3)	18.8 (12.1-25.9)	28.1 (16.4-38.0)	18.3 (9.2-32.4)	16.5 (9.1-28.5)	16.1 (8.4-28.2)
Aged ≥25 y with at least 4 y of college	37.6 (25.2-50.9)	24.9 (16.2-35.1)	22.4 (15.0-33.6)	30.6 (21.0-49.6)	31.2 (20.8-44.9)	27.8 (17.3-43.9)
Median household income (standardized to 0-100)	24.0 (15.6-39.1)	23.8 (16.5-32.4)	18 (13.5-25.6)	22 (16.1-33.0)	22.0 (16.2-29.2)	23.0 (15.4-34.7)
Median value of owner-occupied homes (standardized to 0-100)	29.0 (21.6-35.7)	17.9 (14.6-23.3)	24.6 (15.9-33.7)	28.3 (19.6-35.3)	27.5 (19.0-35.7)	26.5 (19.5-34.4)
Cardiovascular conditions						
Hypertension	627 (59.9)	279 (74.4)	354 (49.5)	95 (52.8)	99 (47.8)	37 (37.4)
Hyperlipidemia	532 (50.8)	160 (42.7)	279 (39.0)	61 (33.9)	62 (30.0)	27 (27.3)
Coronary artery disease	205 (19.6)	35 (9.3)	64 (9.0)	16 (8.9)	23 (11.1)	7 (7.1)
Heart failure	131 (12.5)	43 (11.5)	44 (6.2)	5 (2.8)	9 (4.3)	6 (6.1)
Diabetes	299 (28.6)	161 (42.9)	298 (41.7)	63 (35)	57 (27.5)	34 (34.3)
Cancer	152 (14.5)	43 (11.5)	48 (6.7)	16 (8.9)	21 (10.1)	9 (9.1)
CKD	129 (12.3)	88 (23.5)	100 (14.0)	19 (10.6)	18 (8.7)	5 (5.1)
Temperature, median (IQR), °C	37.3 (36.9-38.1)	37.6 (37.1-38.2)	37.5 (36.9-38.4)	37.3 (36.9-38.1)	37.6 (37-38.3)	37.4 (36.9-38.2)
Temperature, °C						
<38	758 (72.4)	249 (66.4)	462 (64.6)	131 (72.8)	143 (69.1)	65 (65.7)
38-39	198 (18.9)	79 (21.1)	153 (21.4)	29 (16.1)	33 (15.9)	23 (23.2)
>39	90 (8.6)	46 (12.3)	100 (14)	20 (11.1)	31 (15)	10 (10.1)
Oxygenation on arrival, %						
<88	157 (15.0)	53 (14.1)	98 (13.7)	40 (22.2)	42 (20.3)	21 (21.2)
89-92	223 (21.3)	84 (22.4)	196 (27.4)	48 (26.7)	39 (18.8)	17 (17.2)
93-100	666 (63.6)	238 (63.5)	421 (58.9)	92 (51.1)	126 (60.9)	60 (60.6)
Unknown	1 (0.1)	0	0	0	0	1 (1.0)

Abbreviations: BMI, body mass index (calculated as weight in kilograms divided by height in meters squared); CKD, chronic kidney disease; IQR, interquartile range; SES, socioeconomic status.

### Risk of Hospitalization After Testing Positive for COVID-19

In unadjusted models, Hispanic patients were no more likely than White patients to be hospitalized (OR, 1.0; 95% CI, 0.9-1.2; *P* = .74) while Black (OR, 0.7; 95% CI, 0.6-0.9; *P* < .001) and Asian (OR, 0.8; 95% CI, 0.6-0.9; *P* = .03) patients were less likely than White patients to be hospitalized. After adjustment for all covariates, Hispanic (OR, 1.0; 95% CI, 0.8-1.2; *P* = .90) and Black patients (OR, 0.9; 95% CI, 0.7-1.1; *P* = .23) were no more likely than White patients to be hospitalized. After adjusting for age, sex, insurance status, and comorbidity, Asian patients were more likely (OR, 1.5; 95% CI, 1.1-2.0; *P* = .01) than White patients to be hospitalized, and the odds slightly increased with addition of neighborhood SES (OR, 1.6; 95% CI, 1.1-2.3; *P* = .004) to the model (Figure).

**Figure.**
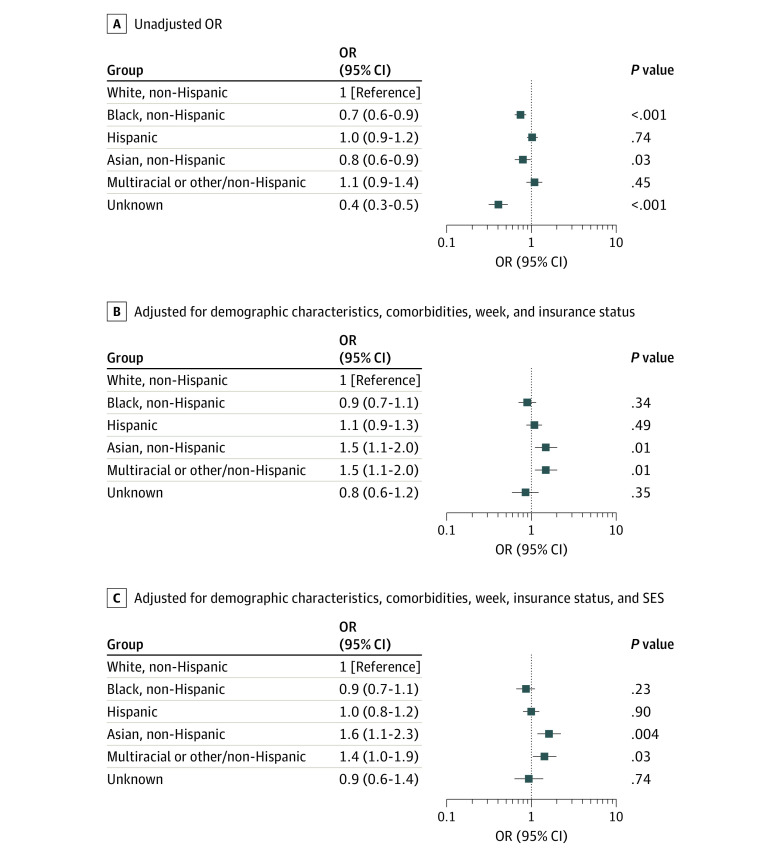
Odds Ratios Comparing Likelihood of Admission by Patient's Race/Ethnicity Among Patients With a Positive COVID-19 Test Result OR indicates odds ratio; SES, socioeconomic status.

### Characteristics of Patients Hospitalized With COVID-19 by Race and Ethnicity

Among the 2623 hospitalized patients (1047 [39.9%] White, 375 [14.3%] Black, 715 [27.3%] Hispanic, 180 [6.9%] Asian, and 207 [7.9%] multiracial/other), neighborhood SES and rates of current smoking and obesity were similar to those reported for patients who tested positive. Hospitalized patients were older and had higher comorbidity compared with patients who tested positive and were not hospitalized. At hospital presentation, Hispanic and multiracial/other patients on average had lower oxygenation on arrival than White and Black patients. Fifty percent of Black patients who were hospitalized were women, whereas other groups had a preponderance of men (range, 59%-74%; Table 4).

**Table 4.** Odds Ratios of Outcomes by Patient Race and Ethnicity

Population	Outcome	Race/ethnicity	Unadjusted		Adjusted^a^		Adjusted^b^	
			OR (95% CI)	*P* value	OR (95% CI)	*P* value	OR (95% CI)	*P* value
All tested patients	COVID-19 positive	White, non-Hispanic	1 [Reference]	>.99	1 [Reference]	>.99	1 [Reference]	>.99
		Black, non-Hispanic	1.6 (1.4-1.8)	<.001	1.6 (1.4-1.9)	<.001	1.3 (1.2-1.6)	<.001
		Hispanic	1.8 (1.6-2.0)	<.001	1.7 (1.5-1.9)	<.001	1.5 (1.3-1.7)	<.001
		Asian, non-Hispanic	0.9 (0.8-1.0)	.05	1 (0.8-1.1)	.70	0.9 (0.8-1.1)	.21
		Multiracial/other, non-Hispanic	1.2 (1.1-1.5)	.004	1.3 (1.1-1.5)	.004	1.1 0.9-1.4)	.15
		Unknown	1 (0.9-1.2)	.87	1.2 (0.9-1.4)	.08	1 (0.9-1.3)	.71
Patients with a positive test result	COVID-19 admission	White, non-Hispanic	1 [Reference]	>.99	1 [Reference]	>.99	1 [Reference]	>.99
		Black, non-Hispanic	0.7 (0.6-0.9)	<.001	0.9 (0.7-1.1)	.34	0.9 (0.7-1.1)	.23
		Hispanic	1.0 (0.9-1.2)	.74	1.1 (0.9-1.3)	.49	1.0 (0.8-1.2)	.90
		Asian, non-Hispanic	0.8 (0.6-0.9)	.03	1.5 (1.1-2.0)	.01	1.6 (1.1-2.3)	.004
		Multiracial/other, non-Hispanic	1.1 (0.9-1.4)	.45	1.5 (1.1-2.0)	.01	1.4 (1.0-1.9)	.03
		Unknown	0.4 (0.3-0.5)	<.001	0.8 (0.6-1.2)	.35	0.9 (0.6-1.4)	.74
Hospitalized patients	Critical illness	White, non-Hispanic	1 [Reference]	>.99	1 [Reference]	>.99	1 [Reference]	>.99
		Black, non-Hispanic	0.6 (0.5-0.8)	<.001	0.7 (0.5-0.9)	.005	0.6 (0.4-0.8)	.001
		Hispanic	0.7 (0.6-0.9)	.001	0.9 (0.7-1.2)	.57	0.9 (0.7-1.2)	.39
		Asian, non-Hispanic	1.1 (0.8-1.5)	.55	1.3 (0.9-1.9)	.10	1.3 (0.9-1.8)	.21
		Multiracial/other, non-Hispanic	0.9 (0.7-1.2)	.56	1.2 (0.9-1.6)	.32	1.2 (0.8-1.8)	.31
		Unknown	0.9 (0.6-1.4)	.69	1.1 (0.7-1.7)	.76	1.0 (0.5-1.5)	.71
	Mortality, HR (95% CI)	White, non-Hispanic	1 [Reference]	>.99	1 [Reference]	>.99	1 [Reference]	>.99
		Black, non-Hispanic	0.6 (0.5-0.8)	<.001	0.7 (0.5-0.9)	.02	0.7 (0.6-0.9)	.04
		Hispanic	0.7 (0.6-0.9)	.001	0.9 (0.7-1.1)	.44	1.0 (0 .8-1.2)	.75
		Asian, non-Hispanic	1.0 (0.7-1.3)	.94	1.2 (0.9-1.7)	.21	1.3 (0.9-1.7)	.16
		Multiracial/other, non-Hispanic	0.7 (0.5-1.0)	.06	0.9 (0.7-1.2)	.52	0.9 (0.7-1.2)	.52
		Unknown	0.8 (0.5-1.2)	.29	1.0 (0.6-1.6)	.95	1.0 (0.6-1.6)	>.99

Abbreviations: HR, hazard ratio; OR, odds ratio.

^a^ Adjusted for demographic characteristics, comorbidities, week, and insurance.

^b^ Adjusted for demographic characteristics, comorbidities, week, insurance, and socioeconomic status (and laboratory results for critical illness outcome).

### Risk of Critical Illness After Hospitalization Among Patients Who Tested Positive for COVID-19

In unadjusted models, Black patients (OR, 0.6; 95% CI, 0.5-0.8) and Hispanic patients (OR, 0.7; 95% CI, 0.6-0.9) had a lower risk of critical illness than White patients. After adjustment for age, sex, insurance status, and comorbidity, the risk of critical illness for Hispanic patients was comparable with White patients, while the risk remained lower in Black patients (OR, 0.7; 95% CI, 0.5-0.9). The addition of neighborhood SES to the model did not appreciably alter the risk of critical illness in Black patients (OR, 0.6; 95% CI, 0.4-0.8). In unadjusted and adjusted models, Asian patients were no more likely than White patients to have critical illness (eg, unadjusted model: OR, 1.1; 95% CI, 0.8-1.5).

### Risk of Death After Hospitalization Among Patients Who Tested Positive for COVID-19

In unadjusted models, Black patients (hazard ratio [HR], 0.6; 95% CI, 0.5-0.8) and Hispanic patients (HR, 0.7; 95% CI, 0.6-0.9) were less likely than White patients to die or be discharged to hospice. After adjusting for age, sex, insurance status, and comorbidity, Black patients continued to have lower risk of death compared with White patients (HR, 0.7; 95% CI, 0.5-0.9). Additional adjustment for severity of illness and neighborhood SES did not alter the lower risk of death in Black patients compared with White patients (HR, 0.7; 95% CI, 0.6-0.9). Hispanic patients (HR, 1.0; 95% CI, 0.8-1.2) and Asian patients (HR, 1.3; 95% CI, 0.9-1.7) had risk of death or discharge to hospice comparable with White patients after adjusting for age, sex, insurance status, comorbidity, and neighborhood SES.

## Discussion

We compared racial/ethnic differences in the likelihood of testing positive for COVID-19, hospitalization, critical illness, and death, and also assessed whether any differences in these outcomes were associated with comorbidity, age, sex, insurance, and neighborhood SES. Black and Hispanic patients were more likely than White patients to test positive for COVID-19. Contrary to our expectation, Black and Hispanic patients were no more likely than White patients to be hospitalized, while Asian patients had higher odds of hospitalization than White patients in adjusted models. In hospitalized patients, Black patients had lower odds of critical illness and death compared with White patients, while Hispanic and Asian patients had comparable odds of critical illness and death compared with White patients.

The higher likelihood of testing positive for COVID-19 among Black and Hispanic patients in our study is consistent with findings from previous studies.^1,2,12,13,24^ This may be because of pervasive social inequalities that increase the difficulty in implementing social distancing in Black and Hispanic communities. Black and Hispanic patients are associated with a lower neighborhood SES and are more likely to work in occupations that are not amenable to working remotely compared with White patients; in New York City, 75% of all frontline workers are people of color.^25,26^ Unfortunately, we do not have complete data on patient occupation and thus were unable to test the role of occupation in the association between race/ethnicity and testing positive for COVID-19.

However, contrary to previous reports,^1,2,12,13,24,25^ Black and Hispanic patients in our study were no more likely than White patients to be hospitalized once they tested positive. In a study of 3481 COVID-19 positive patients (70% Black and 30% non-Hispanic White patients) from an integrated health system in Louisiana,^12^ Black patients were twice as likely as White patients to be hospitalized after adjusting for age, sex, comorbidity, insurance, obesity, and residence in a low-income neighborhood. Similarly, in a study of 1052 patients (5.8% Black, 25.8% Hispanic, and 40.0% White) from an integrated health system in northern California, Azar et al^20^ showed that Black patients were 2.7 times more likely than White patients to be hospitalized after adjusting for comorbidity, sex, age, and median neighborhood household income.

The comparable odds of hospitalization between Black and White patients in our study suggest that both groups had timely access to diagnostic testing and care and comparable severity of COVID-19 illness at presentation. This may relate to insurance status and improved access to care: in the Louisiana study,^12,24,25^ 15% of Black patients received insurance through Medicaid and 9% were uninsured, compared with 5% and 9%, respectively, for White patients. In our cohort, 10% of Black patients testing COVID-19 positive received insurance through Medicaid and 5% were uninsured, both comparable rates with White patients. However, we did not observe higher adjusted admission rates for Hispanic patients, even though they were much more likely to have Medicaid insurance (32%) or lack insurance (6%) compared with White patients. Other factors such as patient preferences (ie, patients may have been admitted but refused because of mistrust of the health care system) and provider bias are difficult to assess because they are variably documented in the medical record.

Black patients in our study had lower odds of critical illness and deaths compared with White patients after adjustment for comorbidity and neighborhood SES. In this regard, our findings are similar to both the Louisiana and California reports, both of which found that Black race was not associated with increased hazard of in-hospital mortality compared with White race.^12,24^ In another study of 305 patients (80% Black) in Georgia,^27,28^ Black patients were also no more likely than White patients to be on invasive mechanical ventilation, to die, or to experience the composite outcome of mechanical ventilation or death. Comparable or even lower rates of adjusted within-hospital mortality in Black and Hispanic patients compared with White patients are also well documented in other diseases.^29,30,31,32^ As with previous studies, our findings clearly showed that if Black patients make it to the hospital, the odds of mortality are either similar or lower than those of White patients, despite having poorer neighborhood SES than White patients. We thus inferred that the higher mortality noted in Black populations could largely be attributed to higher out-of-hospital deaths, which could certainly be explained by the lower neighborhood SES noted in Black communities. Recent data from our group point to the fact that Black residents of poorer zip codes have 3 times higher rates of mortality than their White counterparts.^33^

What could explain the stark contrast between our (and others’) findings of lower odds of critical illness and mortality among hospitalized patients and the well-established overall higher risk of death from COVID-19 disease in Black and Hispanic people in the general population, including in NYC?^1,2,12,27^ The higher rate of COVID-19–related mortality reported for Black populations has been attributed to several factors. First, as noted above, Black and Hispanic patients may be disproportionately likely to contract COVID-19 disease, which could increase absolute death rates even if there were no difference in mortality risk once testing positive. Second, Black populations are more likely to be uninsured and underinsured than White populations and thus have poorer access to care, which may lead to deaths at home that are not observed in studies of hospitalized patients.^34,35^ Indeed, our cohort of Black and Hispanic patients testing positive for COVID-19 had higher rates of being uninsured than White patients (5.1% and 6.2% vs 2.9%), which is similar to the overall NYC/Nassau County population (6.1% of Black, 3.3% of White, and 11.3% of Hispanic patients with no insurance). However, Black and Hispanic patients in our hospitalized cohort were much less likely to be uninsured (1.3% and 5.0%) compared with the Black and Hispanic populations overall in NYC. It is possible, therefore, that uninsured patients disproportionately avoided the hospital when more acutely ill, leading to undetected deaths at home and contributing to higher mortality rates among Black and Hispanic populations from COVID-19 in the general population. Possible reasons for avoiding the hospital may include fear of acquiring COVID-19 in the hospital or misperceptions of the signs or symptoms that require medical attention. Third, a strong predictor of poor COVID-19 outcomes in hospitalized patients is male sex.^8^ The proportion of Black patients who are female (62.1%) in our study is higher than that of other groups (most <50%), perhaps explaining their relatively better outcomes, though we did adjust for sex in our analyses. The Louisiana study also demonstrated a disproportionate fraction of women among Black patients (62.3% vs 54.3% for White patients). Perhaps Black men are more likely to avoid care, producing worse outcomes out of the hospital. Fourth, Black and Hispanic populations may disproportionately receive care in less well-resourced hospital settings, worsening overall outcomes, even if within-hospital outcomes are the same for Black and White patients.^4^ Fifth, our study population may not be representative of the overall New York City population.^9,13^ In the study by Wadhera et al,^13^ the Bronx had the highest rate of COVID-19 related hospitalization and mortality. Our catchment area draws more from Manhattan, Brooklyn, Queens, and Long Island. For instance, the mean neighborhood SES index of the patients with COVID-19 in our population was 53.7 compared with a population-weighted mean of 48.3 for NYC and Long Island, although not all of our patients are from those areas.

### Strengths and Limitations

The strengths of our study include a large sample size, a high follow-up rate, and full adjustment of our findings for insurance status, neighborhood SES, and comorbidity using data from an integrated health system. Unlike prior studies, we adjusted for effects of crowding, educational status, and unemployment. Other prior studies are largely based on publicly available data sets that do not account for individual patient comorbidity and insurance status.

This study had the following limitations. Some patients who tested positive at NYULH could have been hospitalized subsequently at another medical center. However, we have no reason to believe this would differ markedly by race or ethnicity. We did not have complete data on the final outcome of 4% of hospitalized patients, although this study had better follow up than other COVID-19 case series.^6^

## Conclusions

Our findings support the notion that Black and Hispanic populations are not inherently more susceptible to having poor COVID-19 outcomes than other groups and, more importantly, that if they make it to the hospital they fare as well as or better than their White counterparts. This supports the assertion that existing structural determinants—including inequality in housing, access to care, differential employment opportunities, and poverty—that remain pervasive in Black and Hispanic communities should be addressed in order to improve outcomes in COVID-19–related mortality. Future research should explore the direct impact of structural inequities on racial and ethnic disparities in COVID-19 related hospitalization, morbidity, and mortality.

## Notes

Supplement. **eFigure.** Study Flow Chart **eTable.** Overall Demographic Characteristics of the Cohort

## References

[1] Artiga SG, Garfield R, Orgera K Communities of color at higher risk for health and economic challenges due to COVID-19. Kaiser Family Foundation April 7, 2020 Accessed October 26, 2020. https://www.kff.org/coronavirus-covid-19/issue-brief/communities-of-color-at-higher-risk-for-health-and-economic-challenges-due-to-covid-19/#:~:text=Communities%20of%20color%20will%20likely,accessing%20care%20than%20Whites%3B%20and

[2] US Centers for Disease Control and Prevention COVID-19 in Racial and Ethnic Minority Groups. Centers for Disease Control and Prevention; 2020.

[3] Wechkunanukul K, Grantham H, Damarell R, Clark RA The association between ethnicity and delay in seeking medical care for chest pain: a systematic review. JBI Database System Rev Implement Rep. 2016;14(7):208-235. doi:10.11124/JBISRIR-2016-003012 27532797

[4] Hasnain-Wynia R, Baker DW, Nerenz D, Disparities in health care are driven by where minority patients seek care: examination of the hospital quality alliance measures. Arch Intern Med. 2007;167(12):1233-1239. doi:10.1001/archinte.167.12.1233 17592095

[5] Emami A, Javanmardi F, Pirbonyeh N, Akbari A Prevalence of underlying diseases in hospitalized patients with COVID-19: a systematic review and meta-analysis. Arch Acad Emerg Med. 2020;8(1):e35. 32232218 PMC7096724

[6] Richardson S, Hirsch JS, Narasimhan M, ; the Northwell COVID-19 Research Consortium Presenting characteristics, comorbidities, and outcomes among 5700 patients hospitalized with COVID-19 in the New York City area. JAMA. 2020;323(20):2052-2059. doi:10.1001/jama.2020.6775 32320003 PMC7177629

[7] Wang B, Li R, Lu Z, Huang Y Does comorbidity increase the risk of patients with COVID-19: evidence from meta-analysis. Aging (Albany NY). 2020;12(7):6049-6057. doi:10.18632/aging.103000 32267833 PMC7185114

[8] Petrilli CM, Jones SA, Yang J, Factors associated with hospital admission and critical illness among 5279 people with coronavirus disease 2019 in New York City: prospective cohort study. BMJ. 2020;369:m1966. doi:10.1136/bmj.m1966 32444366 PMC7243801

[9] Yancy CW COVID-19 and African Americans. JAMA. 2020;323(19):1891-1892. doi:10.1001/jama.2020.6548 32293639

[10] Owen WF Jr, Carmona R, Pomeroy C Failing another national stress test on health disparities. JAMA. 2020;323(19):1905-1906. doi:10.1001/jama.2020.6547 32293642

[11] Corl K, Levy M, Phillips G, Terry K, Friedrich M, Trivedi AN Racial and ethnic disparities in care following the New York State sepsis initiative. Health Aff (Millwood). 2019;38(7):1119-1126. doi:10.1377/hlthaff.2018.05381 31260359 PMC6814952

[12] Price-Haywood EG, Burton J, Fort D, Seoane L Hospitalization and mortality among black patients and white patients with COVID-19. N Engl J Med. 2020;382(26):2534-2543. doi:10.1056/NEJMsa2011686 32459916 PMC7269015

[13] Wadhera RK, Wadhera P, Gaba P, Variation in COVID-19 hospitalizations and deaths across New York City boroughs. JAMA. 2020;323(21):2192-2195. doi:10.1001/jama.2020.7197 32347898 PMC7191469

[14] NYCDOHMH Cases, Hospitalizations and Deaths. New York City Department of Health and Mental Hygiene; 2020.

[15] Reitzel LR, Regan SD, Nguyen N, Density and proximity of fast food restaurants and body mass index among African Americans. Am J Public Health. 2014;104(1):110-116. doi:10.2105/AJPH.2012.301140 23678913 PMC3910025

[16] Bonito AJ, Bann C, Eicheldinger C, Carpenter L Chapter 1: creation of new race-ethnicity codes and socioeconomic status (SES) indicators for Medicare beneficiaries. Agency for Healthcare Research and Quality. January 2008 Accessed November 11, 2020. http://archive.ahrq.gov/research/findings/final-reports/medicareindicators/medicareindicators1.html

[17] Blum AB, Egorova NN, Sosunov EA, Impact of socioeconomic status measures on hospital profiling in New York City. Circ Cardiovasc Qual Outcomes. 2014;7(3):391-397. doi:10.1161/CIRCOUTCOMES.113.000520 24823956 PMC4072036

[18] Bonito AJ, Bann C, Eicheldinger C, Carpenter L Creation of new race-ethnicity codes and socioeconomic status (SES) indicators for Medicare beneficiaries: final report. Agency for Healthcare Research and Quality Revised August 2012 Accessed June 4, 2020. https://archive.ahrq.gov/research/findings/final-reports/medicareindicators/index.html

[19] Grey RJ A class of K-sample tests for comparing the cumulative incidence of a competing risk. Ann Stat. 2019;16:1141-1154. doi:10.1214/aos/1176350951

[20] Sfumato P. Goodness-of-fit for time-to-event data. Published 2020 Accessed May 5, 2020. https://www.rdocumentation.org/packages/goftte/versions/1.0.5

[21] Fox J, Monette G. Generalized collinearity diagnostics. J Am Stat Assoc. 1992;87:178-183. doi:10.1080/01621459.1992.10475190

[22] Venables WN, Ripley BD Modern Applied Statistics with S-PLUS. Springer Science & Business Media; 2013.

[23] Venzon D, Moolgavkar S. A method for computing profile-likelihood-based confidence intervals. J Royal Stat Soc: Series C (Applied Statistics). 1988;37(1):87-94. doi:10.2307/2347496

[24] Azar KMJ, Shen Z, Romanelli RJ, Disparities in outcomes among COVID-19 patients in a large health care system in California. Health Aff (Millwood). 2020;39(7):1253-1262. doi:10.1377/hlthaff.2020.00598 32437224

[25] Webb Hooper M, Nápoles AM, Pérez-Stable EJ COVID-19 and racial/ethnic disparities. JAMA. 2020;323(24):2466-2467. doi:10.1001/jama.2020.8598 32391864 PMC9310097

[26] Stringer SM New York City's frontline workers. Published March 26, 2020 Accessed June 4, 2020. https://comptroller.nyc.gov/reports/new-york-citys-frontline-workers/

[27] Gold JAW, Wong KK, Szablewski CM, Characteristics and clinical outcomes of adult patients hospitalized with COVID-19—Georgia, March 2020. MMWR Morb Mortal Wkly Rep. 2020;69(18):545-550. doi:10.15585/mmwr.mm6918e1 32379729 PMC7737948

[28] Yehia BR, Winegar A, Fogel R, Association of race with mortality among patients hospitalized with coronavirus disease 2019 (COVID-19) at 92 US hospitals. JAMA Netw Open. 2020;3(8):e2018039. doi:10.1001/jamanetworkopen.2020.18039 32809033 PMC7435340

[29] Barnato AE, Lucas FL, Staiger D, Wennberg DE, Chandra A Hospital-level racial disparities in acute myocardial infarction treatment and outcomes. Med Care. 2005;43(4):308-319. doi:10.1097/01.mlr.0000156848.62086.06 15778634 PMC2121607

[30] Downing NS, Wang C, Gupta A, Association of racial and socioeconomic disparities with outcomes among patients hospitalized with acute myocardial infarction, heart failure, and pneumonia: an analysis of within-and between-hospital variation. JAMA Netw Open. 2018;1(5):e182044. doi:10.1001/jamanetworkopen.2018.2044 30646146 PMC6324513

[31] Hasnain-Wynia R, Kang R, Landrum MB, Vogeli C, Baker DW, Weissman JS Racial and ethnic disparities within and between hospitals for inpatient quality of care: an examination of patient-level Hospital Quality Alliance measures. J Health Care Poor Underserved. 2010;21(2):629-648. doi:10.1353/hpu.0.0281 20453362

[32] Thomas KL, Hernandez AF, Dai D, Association of race/ethnicity with clinical risk factors, quality of care, and acute outcomes in patients hospitalized with heart failure. Am Heart J. 2011;161(4):746-754. doi:10.1016/j.ahj.2011.01.012 21473975

[33] Adhikari S, Pantaleo NP, Feldman JM, Ogedegbe O, Thorpe L, Troxel AB Assessment of community-level disparities in coronavirus disease 2019 (COVID-19) infections and deaths in large US metropolitan areas. JAMA Netw Open. 2020;3(7):e2016938-e2016938. doi:10.1001/jamanetworkopen.2020.16938 32721027 PMC7388025

[34] Williams DR, Jackson PB Social sources of racial disparities in health. Health Aff (Millwood). 2005;24(2):325-334. doi:10.1377/hlthaff.24.2.325 15757915

[35] Levine RS, Foster JE, Fullilove RE, Black-white inequalities in mortality and life expectancy, 1933–1999: implications for healthy people 2010. Public Health Rep. 2001;116(5):474-483. doi:10.1093/phr/116.5.474 12042611 PMC1497364

