# Supplementary material for: Assessment of Racial/Ethnic Disparities in Hospitalization and Mortality in Patients With COVID-19 in New York City
Source: JAMA Netw Open. 2020 Dec 4;3(12):e2026881. doi: 10.1001/jamanetworkopen.2020.26881 (PMC7718605; doi:10.1001/jamanetworkopen.2020.26881)
Supplement: Supplement. — eFigure. Study Flow Chart eTable. Overall Demographic Characteristics of the Cohort [file jamanetwopen-e2026881-s001.pdf]

Supplemental Online Content

Ogedegbe G, Ravenell J, Adhikari S, et al. Assessment of racial/ethnic disparities in hospitalization and mortality in patients with COVID-19 in New York City. *JAMA Netw Open*. 2020;3(12):e2026881. doi:10.1001/jamanetworkopen.2020.26881

eFigure. Study Flow Chart
eTable. Overall Demographic Characteristics of the Cohort

This supplemental material has been provided by the authors to give readers additional information about their work.

© 2020 Ogedegbe G et al. *JAMA Network Open.*

eFigure. Study Flow Chart

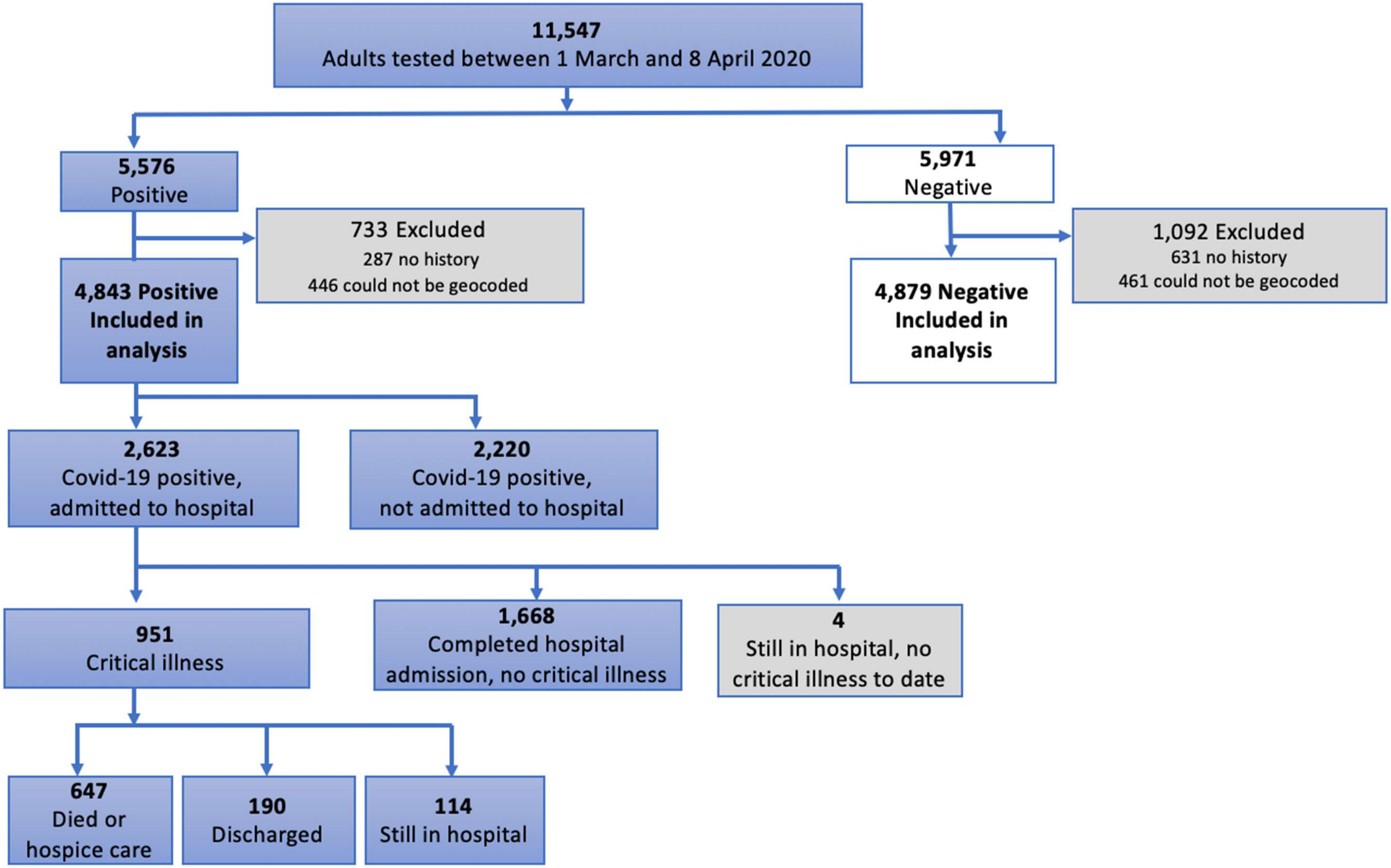

© 2020 Ogedegbe G et al. *JAMA Network Open.*

eTable. Overall Demographic Characteristics of the Cohort

Characteristics	Detected<br>n = 4843	Not-Detected<br>n = 4879	Overall<br>n = 9722
**Age, n(%)**			
19-44	1594 (32.9)	2442 (50.0)	4036 (41.5)
45-54	829 (17.1)	855 (17.5)	1684 (17.3)
55-64	976 (20.1)	823 (16.9)	1799 (18.5)
65-74	758 (15.7)	392 (8.0)	1150 (11.8)
>=75	686 (14.2)	367 (7.5)	1053 (10.8)
**Gender, n(%)**			
Female	2428 (50.1)	3289 (67.4)	5717 (58.8)
Male	2415 (49.9)	1590 (32.6)	4005 (41.2)
**Insurance, (%)**			
Commercial	2401 (49.6)	3402 (69.7)	5803 (59.7)
Medicaid	783 (16.2)	367 (7.5)	1150 (11.8)
Medicare	1269 (2.2)	677 (13.8)	1946 (20.0)
Other	162 (3.3)	105 (2.1)	267 (2.7)
Uninsured/Self-Pay	228 (4.7)	328 (6.7)	556 (5.7)
**Smoking status, n(%)**			
Current	272 (5.6)	503 (10.3)	775 (7.9)
Former	869 (17.9)	770 (15.8)	1639 (16.8)
Never,	3079 (63.6)	3276 (67.1)	6355 (65.4)
Unknown	623 (12.9)	330 (6.8)	953 (9.8)
**BMI, median (IQR)**	28.73 [25.1, 33.4]	27.3 [23.5, 31.6]	28.2 [24.5, 32.64]
**BMI group, n(%)**			
BMI, <25	1275 (26.3)	1886 (38.6)	3161 (32.5)
BMI, 25-30	1633 (33.7)	1504 (30.8)	3137 (32.3)
BMI, 31 - 40	1461 (30.2)	1142 (23.4)	2603 (26.8)
BMI, >40	300 (6.2)	200 (4.1)	500 (51.4)
Unknown	174 (3.6)	147 (3.0)	321 (3.3)
**SES index, median (IQR)**	53.7 [49.1, 58.0]	56.1 [51.8, 60.2]	54.9 [50.3, 59.3]

© 2020 Ogedegbe G et al. *JAMA Network Open.*

IQR= Inter Quartile Range

SES= Socioeconomic status

BMI= Body Mass Index

© 2020 Ogedegbe G et al. *JAMA Network Open.*
